# Monarchs Reared in Winter in California Are Not Large Enough to Be Migrants. Comment on James et al. First Population Study on Winter Breeding Monarch Butterflies, *Danaus plexippus* (Lepidoptera: Nymphalidae) in the Urban South Bay of San Francisco, California. *Insects* 2021, *12*, 946

**DOI:** 10.3390/insects13010063

**Published:** 2022-01-06

**Authors:** Andrew K. Davis

**Affiliations:** Odum School of Ecology, University of Georgia, Athens, GA 30602, USA; akdavis@uga.edu

## 1. Synopsis

A recent study in this journal aimed to understand certain changes in the wintering behavior of monarch butterflies, specifically in the western subpopulation of North America. In the winter of 2020/2021, the number of monarchs arriving at historical colonies in California dropped precipitously; meanwhile reports of larval monarchs during that winter increased. The proliferation of non-native milkweed at sites in California, along with gradually rising temperatures, are thought to have triggered traditionally-migratory monarchs to become reproductive during the winter months. Whether these monarchs or their offspring resume their migratory behavior is a critical question. James et al. (2021) reared 500 larval monarchs from such sites and released them with wing tags to track their movement [1]. Based on the fact that a smaller number were recovered during the spring, and they appeared to have a northward trajectory, they concluded that these monarchs rejoined the migratory cohort on their return northward. This implies that these non-native milkweed sites can provide monarchs with an alternative wintering strategy, and, that monarchs that develop in such areas can bolster the migratory cohort. Here, I contest this claim by showing how the monarchs reared in that study were smaller than traditionally-migratory western monarchs, and in fact resembled the size of every other non-migratory population of monarchs studied. This suggests that these larvae were likely the offspring of local (non-migratory) resident monarchs, which have been proliferating thanks to the planting of non-native milkweed. Moreover, these would themselves likely become part of the resident population, and not contribute to the migratory cohort.

## 2. Background

The conservation of monarch butterflies in North America has been a topic of considerable interest in recent years, driven largely by declines in the size of their wintering colonies in both the eastern and western subpopulations [2,3]. The situation in western North America appears most pronounced, and in the winter of 2020/2021 there was a precipitous drop in counts of monarchs at the traditional colony sites along the California coastline [4,5]. In that same winter, there were notable increases in sightings of adult monarchs in backyard gardens in coastal sites in California, as well as increases in sightings of breeding activity during the winter months. At these sites, homeowners have increasingly been planting non-native tropical milkweed, *Asclepias curassavica*, which remains in leaf year-round in places where it does not freeze [4,6]. This strategy is controversial, as this plant is known to interfere with the migratory patterns of monarchs in eastern North America [7,8]. At the California sites, the presence of abundant non-native milkweed in the winter, along with the mild weather that winter [5], were thought to have led the western migratory monarchs (which traditionally have been in reproductive diapause during winter) to enter a reproductive state and join with the growing number of resident monarchs at these sites. There is prior evidence of this happening, in fact, where a small number of migratory adults have been found ovipositing on exotic milkweed at such sites in winter [9]. If this happened on a larger scale in the winter of 2020/2021, it would suggest that the formerly-migratory monarchs then became breeders and contributed to the abundance of larval stages observed that winter on the non-native milkweed.

This abrupt transition in winter behavior did not go unnoticed among the community of scientists who study monarchs [4,5]. A recent paper in this journal [1] describes a further investigation into the nature of these winter-breeding monarchs in California. The study, conducted during January through June of 2021, was undertaken to determine whether the winter-breeding monarchs contribute to the overall western population, or if it is a sink; in other words, do the winter-breeding monarchs return to a migratory state once the spring arrives? This question is paramount in the development of effective conservations strategies for the western subpopulation. If this idea holds true, then these local sites in California with abundant tropical milkweed have value as an “alternate wintering strategy for monarchs,” according to the authors [1]. To get at this question, James and three citizen scientist colleagues reared 500 larval monarchs from backyards in the San Francisco Bay area during the winter; from the adults, they tested each for the parasite OE [10], tagged them with numbered stickers for tracking their movements, then reported the numbers of recoveries and the movement of these tagged monarchs. They also provided information on the forewing size of the 500 adult monarchs (see below for details).

There were monarchs released across a period of six months (January–June), and they reported that some tagged monarchs were recovered (by citizens) ~1–2 km away from the release sites. Importantly, the authors reported that during the spring months (March and April), which are the months when traditional winter colonies begin breaking up and preparing for northward migration, there were slightly fewer monarchs recovered, though this finding was not statistically significant (chi-square test, *p* = 0.439). They reported that the direction of “flights” of these monarchs was largely northward, though there were no statistical tests performed to determine if these directions differed from random. Finally, they reported that the overall recovery distance was farthest during April. James et al. interpreted these results as evidence that many of the monarchs reared in winter likely left the area in the spring, thereby implying that they became migratory and/or then contributed to the larger migratory cohort. They also used later reports of scattered adult monarchs found in Oregon and Washington in the summer of 2021 as indirect evidence that the monarchs reared in winter in the Bay area contributed to this migratory cohort. Collectively, James et al. (2021) used these pieces of evidence to form a narrative that implies that the sites in California with abundant non-native milkweed provide a temporary refuge for the migratory western monarchs, and therefore that this practice of providing non-native milkweed for winter breeding is an effective conservation strategy for the western subpopulation [1]. 

The James et al. study is timely and certainly has value in helping to understand this recent biological phenomenon; however, I contend that the authors have extrapolated too much from their findings, and then drawn erroneous conclusions regarding the conservation value of these sites in California. For example, the authors interpreted the slightly lower tag recovery rate in the spring months (12–13%) and the slightly longer recovery distance in April (2 km on average) as evidence of dispersal away from the sites, which they conflate with northward migration. However, in a prior study from this same lab, any tagged monarchs recovered within 10 km of the release point were not considered evidence of migration [9]. This in fact seems like a reasonable cutoff; tagging data from non-migratory monarchs in New Zealand showed that movements between 1 and 10 km are common [11], and similarly, non-migratory monarchs in Australia travel between 6 and 15 km [12]. Zalucki et al. (2016) determined through modelling that breeding monarchs typically move ~12 km in their lifetime [13]. Thus, all of the evidence indicates movements of “many kilometers” are common for breeding monarchs. This then implies that the 1–2 km (average) flight distances observed by James et al. are unlikely to represent migratory flights. Moreover, even the “low” tag recovery rate observed by James et al. in the spring is on par with that found in non-migratory monarchs elsewhere, 12% [11]. Aside from these misinterpreted findings, a critical piece of information was overlooked in the James et al. paper, which I explain below.

## 3. Alternative Interpretation

For most migratory animals, having a large wing size is important for effectively completing their long-distance journey; this is true of birds [14] and bats [15] as well as of insects, including dragonflies [16] and of course, monarchs. Monarchs have been especially well-studied throughout the world, where there are many introduced populations that have largely adopted a non-migratory lifestyle where conditions preclude the need for long-distance migration [17]. As such, there are resident populations in the Pacific islands, the Caribbean, and even New Zealand. One common feature of these resident monarchs is that they are smaller than those in the traditional North American population, a finding that has been consistently reported in cross-population studies [18,19,20].

To illustrate this point, I compiled a non-exhaustive list of published studies where the forewing lengths of monarchs were measured across different populations and subpopulations around the world (Table 1). For simplicity, I only included one study that reports wing size of the eastern North America subpopulation [18], even though there are many to choose from. I included multiple studies conducted on western monarchs which were based on samples collected at traditional overwintering colonies in California (i.e., truly migratory monarchs). Then, I included studies that measured known non-migratory monarch populations in locations such as Hawaii, Cuba, Spain, etc. Finally, the reported wing sizes of the 500 winter-reared monarchs from James et al. were included for comparison.

**Figure 1:**
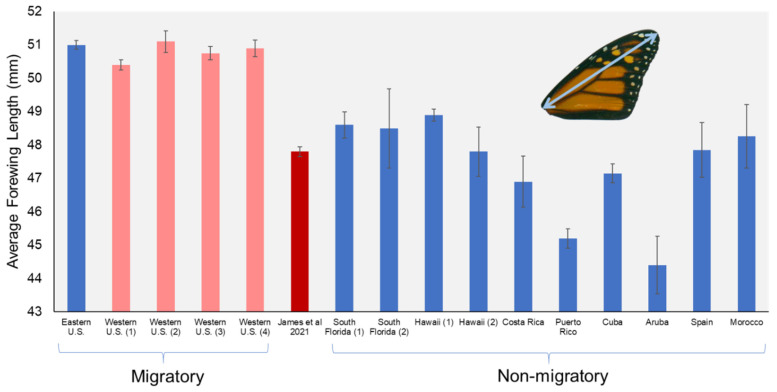
Graph showing mean forewing lengths of monarch butterflies from around the world, (blue bar) based on published sources, for comparison with data presented in James et al., 2021 (red bar). Pink bars represent collections of traditional migratory monarchs during winter in California. See Table 1 for sources and sample sizes of each bar/population. Whiskers around means represent standard errors.

When the wing sizes of all collections are plotted side by side (Figure 1), it becomes obvious that the traditionally migratory western North American monarchs have wings that are as large as those from the east, which is consistent with the current evidence that eastern and western monarchs are not dissimilar [24,25], and that the demands of long-distance migration select for large wing size [26,27]. Meanwhile, the monarchs reared by James et al. had an average wing length that more closely matches those from non-migratory populations (Figure 1). In fact, the average wing length reported by James et al., 47.8 mm, is nearly identical to the average of all non-migratory samples compiled here (47.4 mm; Table 1). Therefore, the fact that the winter-reared California larvae became small-winged adults argues that these monarchs did not develop into migratory individuals. If they were offspring of migrants and had retained their migratory nature, their wing morphology would have more closely matched that of traditional overwintering adults in California. This seems clear, as monarchs are not known to display inter-generational differences in wing design or shape, as seen in some butterflies [28]. By extension, then, one could argue that these monarchs likely did not migrate northward and re-join the migratory cohort, and if they did, then they did not bolster the traditional western monarch subpopulation. Alternatively, even if these winter-reared monarchs developed with the right “instincts” and urge to be migratory adults, their small wings would handicap them from the start, and they would therefore have a reduced likelihood of successful northward migration in the spring. Regardless of which scenario is the case, the evidence regarding the wing size of the winter-reared monarchs is damning to the argument made by James et al., i.e., that the local sites in California with abundant tropical milkweed provides a boost to the migratory population.

I would argue that the small wings of monarchs in the James et al. study reflected the fact that the larvae were offspring of the growing resident (non-migratory) population of the area, which themselves likely had small wings as the result of relaxed selection pressure. Likely, these offspring and others like them would simply end up remaining in the local population, thereby not contributing to the migratory cohort. Of course, there are other possible explanations, which I review here. First, the fact that the monarchs were reared in captivity could be a factor in their small wings, as this is known to lead to small wing size in monarchs [29]. Second, these monarchs were reportedly heavily infected with the parasite *Ophryocystis elektroscirrha* (OE), which is known to reduce wing size in monarchs [10]. The authors indicated that 73% of the monarchs were infected, which is consistent with prior samples of resident monarchs in California [30]. In fact, the infection alone, regardless of wing size, would be a detriment to long-distance migration, as it is known to hinder flight [31] and result in brittle wings [32]. Finally, it is possible that the monarchs were smaller because they were reared on the non-native tropical milkweed, which is known to produce adult monarchs with blunt wings [33]. In fact, given that all three of these scenarios were documented in this study (captive-reared monarchs, raised on tropical milkweed, and heavily infected), it is easy to make the case that each of these factors contributed to a degree. 

## 4. Implications for Conservation

The James et al. study concluded that the growing region in California that hosts year-round exotic milkweed can have “conservation value” as a site where migratory monarchs can adopt an alternative winter lifestyle (i.e., become reproductive). While this idea seems tempting to embrace for home-gardeners, it hinges on the idea that formerly-migratory monarchs (or their offspring) can resume their migratory nature when conditions warrant it. Otherwise, the switch to residency is permanent, and these sites will become sinks for migrants. As this latter scenario would actually result in the reduction of migratory individuals over time (especially if the region with exotic milkweed grows), this would ultimately undermine conservation efforts targeting the traditional migratory subpopulation. This biological question is only beginning to be addressed with monarchs [34], and thus far it remains unresolved. Therefore, given the potential harm that can be done to this already-imperiled subpopulation, it seems risky to promote this unproven strategy.

While the authors of the James et al. study can be commended for taking swift action to study this rapidly-evolving situation in California, their interpretation of their own findings is in question here. They contend that monarch larvae reared on non-native milkweed in the winter in California and released as adults can contribute to the traditionally migratory population based on non-significant results, circumstantial evidence, and/or no statistical tests at all. Meanwhile, their own data show that the monarchs they reared became, on average, small-winged adults, which is consistent with all other non-migratory populations studied, and, that a large majority of these monarchs were heavily infected with a debilitating pathogen. Thus, even if these monarchs did perceive the correct environmental triggers for commencing spring migration, which is unclear, they would be handicapped from the start even if they attempted to migrate northward along with their wild counterparts. Given that the reared and tagged monarchs were only found within a few kilometers of the release sites, it seems unlikely that they ever attempted migration; therefore, they did not contribute to the migratory cohort, and the conservation value of the local sites in California with abundant non-native milkweed is not resolved by the study of James et al.

## Figures and Tables

**Table 1:** Summary of published data on monarch wing lengths from around the world for comparison with James et al., 2021, who reared monarchs in winter in California. Mean forewing lengths (mm) are presented for each sample; males and females are pooled.

Location	Status	N	Forewing Length	SE	Source
Eastern U.S.	Migratory	389	51.0	0.13	[18]
Western U.S. (1)	Migratory	251	50.4	0.15	[18]
Western U.S. (2)	Migratory	59	51.1	0.32	[19]
Western U.S. (3)	Migratory	242	50.8	0.20	[21]
Western U.S. (4)	Migratory	728	50.9	0.25	[22]
Winter-reared in California		499	47.8	0.15	[1]
South Florida (1)	Nonmigratory	54	48.6	0.40	[18]
South Florida (2)	Nonmigratory	20	48.5	1.19	[19]
Hawaii (1)	Nonmigratory	125	48.9	0.18	[18]
Hawaii (2)	Nonmigratory	24	47.8	0.74	[19]
Costa Rica	Nonmigratory	20	46.9	0.77	[18]
Puerto Rico	Nonmigratory	58	45.2	0.29	[18]
Cuba	Nonmigratory	135	47.2	0.28	[23]
Aruba	Nonmigratory	26	44.4	0.86	[19]
Spain	Nonmigratory	16	47.9	0.82	[19]
Morocco	Nonmigratory	24	48.3	0.96	[19]
Average Migratory		1669	50.8	0.21	
Average Nonmigratory		502	47.4	0.60	

## Data Availability

All data presented here are provided within the article itself. Moreover, all data came from published literature.
